# Inhibition of tumor necrosis factor receptor 1 and the risk of periodontitis

**DOI:** 10.3389/fimmu.2023.1094175

**Published:** 2023-02-09

**Authors:** Zoheir Alayash, Sebastian-Edgar Baumeister, Birte Holtfreter, Thomas Kocher, Hansjörg Baurecht, Benjamin Ehmke, Stefan Lars Reckelkamm, Michael Nolde

**Affiliations:** ^1^ Institute of Health Services Research in Dentistry, University of Münster, Münster, Germany; ^2^ Department of Restorative Dentistry, Periodontology, Endodontology, and Preventive and Pediatric Dentistry, University Medicine Greifswald, Greifswald, Germany; ^3^ Department of Epidemiology and Preventive Medicine, University of Regensburg, Regensburg, Germany; ^4^ Clinic for Periodontology and Conservative Dentistry, University of Münster, Münster, Germany

**Keywords:** tumor necrosis factor (TNF), periodonditis, cis-Mendelian randomization analysis, genetics, proinflammatory cytokine

## Abstract

**Aim:**

To investigate the effect of genetically proxied inhibition of tumor necrosis factor receptor 1 (TNFR1) on the risk of periodontitis.

**Materials and methods:**

Genetic instruments were selected from the vicinity of TNFR superfamily member 1A (TNFRSF1A) gene (chromosome 12; base pairs 6,437,923–6,451,280 as per GRCh37 assembly) based on their association with C-reactive protein (N= 575,531). Summary statistics of these variants were obtained from a genome-wide association study (GWAS) of 17,353 periodontitis cases and 28,210 controls to estimate the effect of TNFR1 inhibition on periodontitis using a fixed-effects inverse method.

**Results:**

Considering rs1800693 as an instrument, we found no effect of TNFR1 inhibition on periodontitis risk (Odds ratio (OR) scaled per standard deviation increment in CRP: 1.57, 95% confidence interval (CI): 0.38;6.46). Similar results were derived from a secondary analysis that used three variants (rs767455, rs4149570, and rs4149577) to index TNFR1 inhibition.

**Conclusions:**

We found no evidence of a potential efficacy of TNFR1 inhibition on periodontitis risk.

## Introduction

Periodontitis is a chronic inflammatory disease of the oral cavity that is linked to an imbalanced relationship between a dysbiotic microbiome and the host inflammatory response, progressively leading to the loss of periodontal ligaments and alveolar bone (1). Tumor necrosis factor (TNF) belongs to a superfamily of cytokines with a broad spectrum of physiological and pathological effects (2). TNF is first translated as a transmembrane molecule (mTNF) and then cleaved by a metalloproteinase (TNF converting enzyme) to produce a soluble form of TNF (sTNF). Both mTNF and sTNF induce cellular responses through binding to the two receptors, TNF receptor 1 (TNFR1) and TNF receptor 2 (TNFR2) (3). While the TNF-mediated signaling pathway of the former promotes cell survival, apoptosis, or necrosis, agonism of the latter induces homeostatic signaling (e.g., tissue regeneration, cell proliferation, and cell survival) (4). In periodontal diseases, TNF plays a major role as an osteoclast-stimulating hormone that mediates bone resorption *via* two distinct pathways: receptor activator of NF-ĸB ligand (RANKL) dependent and RANKL independent (5). During a bacterial infection, a variety of cell types that reside in the periodontium produce and secrete TNF (e.g., macrophages, neutrophils, keratinocytes, fibroblasts, natural killer cells, and T and B cells). Consequently, TNF enhances the expression of RANKL on the cell surface of several cell types, which in turn binds to a RANK receptor on the cell membrane of osteoclast precursors to promote the formation of osteoclasts. In addition, TNF directly binds to its own receptors to induce the differentiation of precursor cells into osteoclasts (6). TNF is considered a key biomarker that reveals insights about diagnosis, prognosis, and treatment in periodontal disease. During inflammatory conditions, such as periodontitis, TNF levels increase in the gingival crevicular fluid to promote the degeneration of inflamed periodontal tissues (7). Several clinical trials have shown an association between salivary TNF and periodontal diseases. TNF was elevated in patients who had clinical indicators of periodontitis, suggesting that this biomarker may be useful in a panel of salivary biomarkers that could facilitate the screening, diagnosis, and management of periodontal diseases (8). Also, TNF levels were reduced in response to periodontitis treatment (9, 10). The most recent case definition system of periodontitis recommended the incorporation of biomarkers, measured from saliva and gingival crevicular fluid, to better assess the prognosis of the disease (11). The update of the classification system is still ongoing to incorporate more robust biomarkers in the future guidelines (12). To date, five licensed TNF inhibitors have been used in treating a set of inflammatory diseases: etanercept, infliximab, adalimumab, golimumab, and certolizumab. These molecules inhibit the formation of the ligand-receptor complex between TNF and its receptors TNFR1/2 *via* different mechanisms (13). The use of TNF inhibitors in animal studies has revealed their potential effectiveness in alleviating periodontal inflammation and reducing alveolar bone loss (14). Similarly, human studies suggested that these medications might improve periodontal clinical parameters in patients with periodontal diseases (10, 15, 16). Findings from systematic reviews are controversial where there is no general agreement on whether TNF inhibitors improve periodontal parameters. Some studies have suggested that treating rheumatoid arthritis (RA) with TNF inhibitors not only alleviates joint inflammation and damage but might also improve periodontal disease outcomes (17). However, other studies reported inconsistent results regarding the efficacy of TNF inhibitors in periodontal diseases (18).

Human genetics helped in identifying the role of genes and genetic polymorphisms in the onset and development of periodontitis (19). The causal pathway from genetic components to periodontitis is often dependent on cytokine gene polymorphisms (20, 21). In addition to discovering genetic components that are directly involved in the etiology of diseases, human genetic data has been extensively used in Mendelian randomization (MR) studies to assess the causal relationship between environmental and biological risk factors and disease susceptibility. MR is an instrumental variable (IV) approach that employs single nucleotide polymorphisms (SNPs) as instruments to index a certain risk factor (22). The random allocation of genetic variants and balancing of environmental factors at conception make an MR design less susceptible to confounding. In addition, these variants are assumed to remain unchanged throughout the lifetime, so they are not affected by the outcome. Thus, the inference drawn through an MR analysis will be less likely due to reverse causation (23). Drug target MR is a cis-MR approach that is an extension to the classical MR method and uses druggable protein expression as an exposure (24). Recent years have witnessed a growing research application of this study design in pharmacoepidemiology to reveal drug-repurposing opportunities (25). The application of MR in this context is considered analogous to in-silico trials that provide virtual randomization to study the therapeutic effect of an intervention in a certain population. For instance, the potential efficacy of IL-6 receptor blocker in preventing coronary heart disease was first discovered utilizing the drug target MR approach (26) then validated in clinical trials leading to the development of ziltivekimab, a novel IL-6R inhibiting drug specifically for use in atherosclerotic disease (27). Considering the growing interest in the potential efficacy of anti-rheumatic agents (e.g., TNF inhibitors) in patients with periodontitis (28) the present study aimed to test if blocking TNFR1 would reduce the risk of periodontitis using a drug target MR approach.

## Materials and methods

### Study design

We applied a cis-MR design based on summary statistics from genome-wide association studies (GWAS) to assess the causal effect of TNFR1 inhibition on periodontitis risk. To ensure the validity of the causal inference derived from an MR approach, IVs must fulfill three key assumptions. 1) The relevance assumption: genetic variants, extracted as IVs, should be associated with the exposure; 2) exchangeability assumption: IVs are independent of confounders of the IV-outcome relation; 3) and the exclusion restriction assumption: IVs affect the outcome only through the exposure and not *via* other biological pathways (i.e., no horizontal pleiotropic effect) (24). In a classical MR study, genetic variants are selected from throughout the genome, while in drug target MR, cis-acting variants are chosen from the vicinity of a specific gene known to encode a target of interest, typically a protein. Cis-variants most likely influence the biological effects of their corresponding gene, hence, strengthening the validity of the genotype-phenotype association and the relevance assumption. In addition, these variants control the expression of their genes rather than the expression of genes that are located outside the protein-encoding region. Thus, the risk of horizontal pleiotropy would be diminished, minimizing the possibility of violating the exchangeability and exclusion restriction assumptions (26). Finally, the choice of cis-variants nullifies the possibility of reverse causation because the likelihood of the direction of causality from the encoded gene towards the phenotype (e.g., disease) is favored over the direction from the phenotype to the encoded protein (24).

### Indexing TNF inhibition

Selective inhibition of TNFR1 guarantees a targeted intervention, halting the pro-inflammatory cascade of events mediated by TNF while keeping the homeostatic signaling, contributed by TNFR2, untouched (4). It has been found that the blockage of TNFR1 inhibits the production and secretion of inflammatory markers like C-reactive protein (CRP), which is often used as a treatment response biomarker of TNF inhibitors (29). In our study, we restricted the pool of potential IVs to SNPs that are in the vicinity of the TNFR superfamily member 1A (TNFRSF1A) gene (chromosome 12; base pairs 6,437,923–6,451,280 as per GRCh37 assembly), the gene that encodes TNFR1, which is a protein embedded in the cell membrane of inflammatory cells. We selected SNPs proximal to the protein-encoding gene (± 1 kb) to minimize the likelihood of selecting SNPs that affect the outcome through an alternative pathway (horizontal pleiotropy) other than the TNF-TNFR1 mediated pathway. Since CRP is a reliable downstream biomarker for the binding of TNF to TNFR1, we retained the IVs from the TNFRSF1A gene that are associated with CRP levels based on summary statistics reported in GWAS. We set the genome-wide significance threshold at <5x10^-5^ and linkage disequilibrium (LD) clumping at r^2^ < 0.001 to select independent SNPs with the strongest evidence of association with systemic inflammation. This algorithm retained a single SNP, rs1800693, which is known to be associated with the increased expression of Δ6-TNFR, a soluble protein that mimics the activity of TNF antagonists (30). Also, we detected 3 other SNPs (rs767455, rs4149570, and rs4149577) in the targeted gene that are associated with CRP levels and had previously been utilized in studies aiming to evaluate the effect of genetically proxied TNFR1 inhibition on several disease outcomes. In these MR studies, the indexing of TNF-TNFR1 signaling inhibition utilizing these 3 SNPs showed protective effects for diseases where TNF inhibitors have been approved for treatment, and unfavorable outcomes where TNF inhibitors are known to exacerbate the symptoms (31, 32).

### Treatment indexing GWAS

We used genetic association estimates with serum concentration of CRP from a GWAS meta-analysis (N=575,531) of the UK biobank (N=427,367) and the Cohorts for Heart and Aging Research in Genomic Epidemiology consortium (N=148,164). Data in these GWAS was derived from participants of European descent. CRP serum concentration was measured by the standard immune assay as mg/L (33).

### Outcome GWAS

Summary statistics for periodontitis were obtained from the Gene-Lifestyle Interactions in Dental Endpoints consortium. A total of 17,353 participants of European ancestry were classified as clinical periodontitis cases and 28,210 as controls. Periodontitis cases were classified by either the Centers for Disease and Control and Prevention/American Academy of Periodontology (CDC/AAP) or the Community Periodontal Index (CPI) case definition (34).

### Statistical analysis

Data for the exposure and outcome were harmonized to ensure that the IV–outcome association and the IV–exposure association refer to the same effect allele. We tested the relevance assumption *via* the F statistic for the IVs’ association with CRP. We set a cutoff of >10, which is generally acceptable to rule out weak instrument bias (35). First, we utilized the only SNP retained from the statistical selection (rs1800693) in our primary analysis and calculated the Wald ratio to estimate periodontitis risk per a standard deviation decrease in CRP mediated *via* TNFR1-blockade. Second, we considered rs767455, rs4149570, and rs4149577 as IVs for our secondary analysis. These SNPs were in LD with r^2^ ≈ 0.7 in the European 1000 version 3 genomes data. Using more genetic variants will plausibly increase the power of the analysis at the cost of including correlated SNPs through LD and consequently exaggerate the precision of causal effects (36). Thus, we derived causal estimates from a fixed-effects inverse variance weighted (IVW) model accounting for the correlation between genetic variants (37). In an alternative way to compensate for correlation, we extended the IVW method by using principle component analysis (PCA). This method guarantees that all variants contribute to the analysis (38). Principal components in the IVW estimator explained over 99% of the variance in the weighted correlation matrix. All analyses were performed in R version 4.1.2 using TwoSampleMR and MendelianRandomization packages.

### Ethics

All analyses were based on publicly available summary statistics without accessing individual-level data; hence, ethical approval was not required. The included GWAS received informed consent from the study participants and have been approved by pertinent local ethical review boards.

## Results

The F-Statistic for the IVs used in our primary and secondary analysis ranged between 54 and 118, indicating no weak instrument bias (**Table 1**). The Wald estimator from our primary analysis failed to show an effect of TNFR1 inhibition on periodontitis risk (Odds ratio (OR): 1.57, 95% confidence interval (CI): 0.38;6.46) (**Table 2**). In our secondary analysis, using three SNPs as IVs (rs767455, rs4149570, and rs4149577), results from the IVW and the PCA method did not show an association between genetically indexed inhibition of TNF-TNFR1 and risk of periodontitis (OR: 0.57, 95% CI: 0.12;2.57, and OR: 0.57, 95% CI: 0.13;2.63, respectively).

**Table 1:** Descriptive information on the TNFRSF1A variants analyzed in the study and their associations with inflammatory markers and disease outcomes.

Instrumental variables	rs1800693^a^	rs767455^b^	rs4149570^b^	rs4149577^b^
Frequency of EA^c^	0.574	0.571	0.374	0.528
F-statistic	118.805	98.010	54.020	81.996
Associations with the inflammatory marker, β (SE), p-value				
C-reactive protein	0.022 (0.002), 9.56e-27	0.020 (0.002), 2.39e-22	0.015 (0.002), 5.23e-13	0.018 (0.002), 9.60e-19
Associations with the outcome, β (SE), p-value				
Periodontitis	0.010 (0.016), 0.530	0.013 (0.016), 0.405	0.004 (0.016), 0.799	0.006 (0.015), 0.685

EA, effect allele; NEA, non-effect allele.

a SNP used in our primary analysis for the Wald ratio estimation.

b SNPs used in our secondary analysis for causal estimation from the multiplicative fixed-effects model and the principle component analysis method.

c Based on allele frequency reported by the genome-wide association studies of C-reactive protein. Single nucleotide polymorphisms were labeled with respect to GRCh37 reference coordinates.

**Table 2:** Mendelian randomization estimates for the effect of tumor necrosis factor receptor 1 inhibition on periodontitis risk.

Periodontitis				
Exposure		OR	95% CI	P-value
CRP	Wald ratio^a^	1.57	(0.38;6.46)	0.53
	IVW^b^	0.57	(0.12;2.57)	0.46
	IVW using PCA^c^	0.57	(0.13;2.63)	0.55

CI, confidence interval; CRP, C-reactive protein; IVW, inverse variance weighted; MPV, mean platelet volume; OR, odds ratio; PCA, principal component analysis.

a Wald ratio of a single SNP, rs1800693.

b IVW for correlated single nucleotide polymorphisms (SNPs) utilized Wald ratios of 3 SNPs (rs767455, rs4149570, and rs4149577).

c IVW using principal components analyses assumed correlated SNPs and are based on Mendelian randomization models of 2 principal components derived from the genetic associations of 3 correlated SNPs (rs767455, rs4149570, and rs4149577).

## Discussion

In this study, we addressed whether genetically proxied TNFR1 inhibition reduces the risk of periodontitis using a drug target MR approach. Our current findings are derived from a robust study design using the largest GWAS summary data but failed to show an effect of TNFR1 inhibition on reducing periodontitis risk.

During periodontal inflammation the periodontal pathogens or their products translocate into the blood circulation to induce the bone marrow resulting in innate immune training of hematopoietic stem and progenitor cells and leading to a hyper-responsiveness of neutrophils. Studies have shown that therapeutic modulation of neutrophil-mediated inflammation represent a plausible therapeutic target for periodontitis treatment (39). Our analysis proxied the inhibition of neutrophils’ since neutrophil-released mediators of inflammatory tissue damage (e.g. elastase, proteinase 3, myeloperoxidase, and matrix metalloproteinases) modulate the activity of proinflammatory cytokines such as TNF, and interleukins (IL-1β, IL-6, and IL-23). A recent drug target MR method found that the downregulation of IL-6 signaling was associated with reduced odds of periodontitis (40), but our analysis did not find a similar effect for TNFR1 inhibition.

Evidence from our MR study is inconsistent with results from cohort studies that aimed to study if TNF inhibitors improve periodontal clinical outcomes (10, 15, 16). Patients diagnosed with periodontal diseases who administered 200 mg of infliximab showed less bleeding on probing (BOP), lower gingival index (GI) and clinical attachment loss (CAL), and shallower pocket depth (PD) than matched patients and healthy controls (10). Another study that included 40 patients experiencing severe periodontitis showed that subjects assigned to TNF inhibitors showed improvements in BOP, PD, and CAL (15).

It is important to bear in mind that comparing our findings with those from conventional observational studies must be done with caution. First, TNF inhibitors used in the clinical setting inhibit the binding of mTNF and sTNF to both receptors, TNFR1 and TNFR2 (10, 15), but our study indexed the selective inhibition of TNFR1. Although it is documented that TNF promotes bone resorption and periodontal disease progression *via* TNFR1, the non-selective inhibition of TNFR might block additional inflammatory pathways that contribute to the efficacy seen in observational studies (5). Second, there is still limited evidence of the therapeutic efficacy of TNF inhibitors in patients diagnosed with periodontitis without other comorbidities. In fact, observational studies that investigated the association between TNF inhibitors and periodontal clinical parameters included patients diagnosed with periodontitis along with inflammatory diseases (10, 15, 16, 41, 42). However, our MR design infers causal estimates across the general population, not in a specific subpopulation of patients diagnosed with inflammatory diseases (e.g., RA). Third, for the treatment of RA, TNF inhibitors are usually prescribed in combination with disease-modifying anti-rheumatic drugs that might also have a favorable impact on the periodontal clinical parameters (43). In addition, there is a biologically plausible interaction between the two agents (44). Ignoring this phenomenon, as in the case of previous observational studies, may bias the results.

Additionally, similar to our findings, results from a systematic review failed to demonstrate convincing protective effects of TNF inhibitors on periodontal parameters (18). TNF inhibitors are usually prescribed for a specific time period, whereas our study aimed to reveal long-term effect of the blockage of TNFR1 on the risk of periodontitis. Thus, our null results could be either a true lack of efficacy or an average of opposing effects. This phenomenon is of clinical relevance since the treatment duration of TNF inhibitors can be a significant factor that influences the clinical outcomes in periodontal diseases. A systematic review showed contradictory periodontal clinical outcomes depending on the duration of treatment of TNF inhibitors (17). At a follow-up of 6 weeks, patients receiving (infliximab, etanercept, or adalimumab) showed improvements in BOP, GI, and CAL (15, 41). Additional periodontal parameters were improved in a longitudinal study when longer treatment duration was observed (up to 6 months) (16). However, treatment for more than 9 months was associated with higher GI and BOP values (42). This discrepancy can be attributed to patient’s noncompliance or secondary loss of response. The latter scenario is a common observation during the treatment with TNF inhibitors, where the host produces antidrug antibodies, thus halting the impact of the medication (45).

Given the disproportionate burden of periodontitis, a growing body of literature investigates the potential efficacy of immunomodulatory treatments, including TNF inhibitors, in periodontal diseases (46); however, drug development remains hindered by the high cost and failure rates. The creation of drug repurposing methods, like cis-MR methods, helps in selecting promising therapeutic candidates for further study in clinical trials. Limitations to this study need to be acknowledged. First, our analysis was based on few genetic IVs. Second, GWAS utilized in our study were conducted on participants of European ancestry; thus, our null results may not be generalizable to other ethnicities.

In conclusion, this instrumental variable analysis failed to find evidence to support the clinical efficacy of TNF inhibitors in reducing the risk of periodontitis. We anticipate that triangulation of evidence from genetics, observational research and clinical trials will elucidate the role of TNF inhibitors in periodontitis. Future work on alternative cytokines or targets of the neutrophil-mediated inflammation may propose drug candidates that alleviate periodontal diseases and prevent periodontitis associated comorbidities.

## Data availability statement

Publicly available datasets were analyzed in this study. This data can be found here: The CRP GWAS was obtained through https://gwas.mrcieu.ac.uk/datasets/ieu-b-35/. The periodontitis summary data are available at https://data.bris.ac.uk/data/dataset/2j2rqgzedxlq02oqbb4vmycnc2.

## Author contributions

Conception and design, ZA and MN. Development of methodology, ZA, S-EB, HB, and MN. Analysis and interpretation of data (e.g., statistical analysis, biostatistics, computational analysis), ZA, S-EB, SR, and MN. Writing, review, and/or revision of the manuscript, ZA, S-EB, SR, BH, TK, HB, BE, and MN. Administrative, technical, or material support (i.e., reporting or organizing data, constructing databases), ZA. All authors contributed to the article and approved the submitted version.
